# 2-[5-(Benzo[*d*]thia­zol-2-yl)thio­phen-2-yl]benzo[*d*]thia­zole

**DOI:** 10.1107/S1600536810004009

**Published:** 2010-02-06

**Authors:** Kim Potgieter, Peter Mayer, Eric Hosten, Thomas I. A. Gerber

**Affiliations:** a Department of Chemistry, Nelson Mandela Metropolitan University, 6031 Port Elizabeth, South Africa; b Department of Chemistry, Ludiwig-Maximilians University, D-81377 München, Germany

## Abstract

The structure of the title compound, C_18_H_10_N_2_S_3_, consists of a central thio­phene ring and two terminal thia­zole rings. The two S atoms of the thia­zole rings are *trans* to the thio­phene S atom sulfur. The thia­zole rings are approximately coplanar with the thio­phene ring, with dihedral angles of 6.23 (11) and 4.81 (11)° between them. In the crystal, zigzag chains are formed along [010] by weak C—H⋯N inter­actions.

## Related literature

For the synthesis of thio­phene derivatives, see: Kaleta *et al.* (2006 ▶); Minetto *et al.* (2005 ▶); Bayh *et al.* (2005 ▶). For their conformation, see: Alberti *et al.* (1986 ▶); Hagen (1986 ▶); Salman (1982 ▶) and for their applications, see: Seed *et al.* (2003 ▶); Cheylan *et al.* (2006 ▶); Karimian (2009 ▶); Kiryanov *et al.* (2001 ▶); Shi *et al.* (1996 ▶).
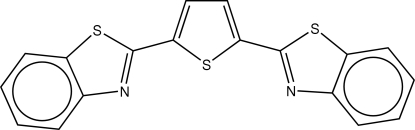


## Experimental

### 

#### Crystal data

C_18_H_10_N_2_S_3_
*M* *_r_* = 350.48
Monoclinic, 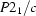

*a* = 15.7297 (14) Å
*b* = 8.2396 (5) Å
*c* = 12.8160 (12) Å
β = 112.872 (11)°
*V* = 1530.4 (2) Å^3^
*Z* = 4
Mo *K*α radiation
μ = 0.48 mm^−1^
*T* = 200 K
0.34 × 0.15 × 0.01 mm

#### Data collection

Oxford XCalibur diffractometer
Absorption correction: multi-scan (*CrysAlis PRO*; Oxford Diffraction, 2009 ▶) *T*_min_ = 0.839, *T*_max_ = 1.000
5789 measured reflections
3086 independent reflections
1452 reflections with *I* > 2σ(*I*)
*R*_int_ = 0.053

#### Refinement

*R*[*F* ^2^ > 2σ(*F* ^2^)] = 0.043
*wR*(*F* ^2^) = 0.051
*S* = 0.72
3086 reflections
208 parameters
H-atom parameters constrained
Δρ_max_ = 0.29 e Å^−3^
Δρ_min_ = −0.30 e Å^−3^

### 

Data collection: *CrysAlis PRO* (Oxford Diffraction, 2009 ▶); cell refinement: *CrysAlis PRO*; data reduction: *CrysAlis PRO*; program(s) used to solve structure: *SIR97* (Altomare *et al.*, 1999 ▶); program(s) used to refine structure: *SHELXL97* (Sheldrick, 2008 ▶); molecular graphics: *ORTEP-3 for Windows* (Farrugia, 1997 ▶) and *Mercury* (Macrae *et al.*, 2006 ▶); software used to prepare material for publication: *publCIF* (Westrip, 2010 ▶) and *PARST* (Nardelli, 1995 ▶).

## Supplementary Material

Crystal structure: contains datablocks I, global. DOI: 10.1107/S1600536810004009/ez2199sup1.cif

Structure factors: contains datablocks I. DOI: 10.1107/S1600536810004009/ez2199Isup2.hkl

Additional supplementary materials:  crystallographic information; 3D view; checkCIF report

## Figures and Tables

**Table 1:** Hydrogen-bond geometry (Å, °)

*D*—H⋯*A*	*D*—H	H⋯*A*	*D*⋯*A*	*D*—H⋯*A*
C16—H16⋯N1^i^	0.95	2.63	3.444 (4)	144

Symmetry code: (i) 
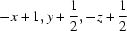
.

**Fig. 1.**
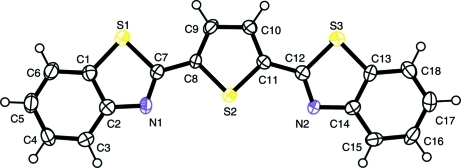
The molecular structure of the title compound (anisotropic displacement ellipsoids drawn at the 50% probablility level).

**Fig. 2.**
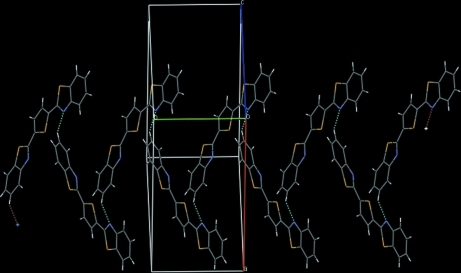
The zig-zag-chains established by weak interactions of the type C–H···N.

## supplementary crystallographic information

### Comment

Derivatives of thiophene have shown promise in photovoltaic (Cheylan *et al.*, 2006), liquid crystal (Kiryanov *et al.* 2001) and therapeutic applications (Seed *et al.*, 2003). On the other hand, thiazole derivatives have been evaluated for biological activity (Karimian, 2009), especially against certain breast carcinoma cell lines (Shi *et al.* 1996).

In the present work the structure of 2-(5-benzo[*d*]thiazol-2-yl)thiophen-2-yl)benzo[*d*]thiazole has been determined to explore its suitability as a tridentate ligand for various metal ions.

The structure consists of a central thiophenyl ring and two terminal thiazolyl rings, with the two sulfur atoms of the latter rings in *trans* positions to the thiophenyl sulfur atom (see Fig. 1). The thiazolyl rings with S1 and S3 are approximately coplanar with the thiophenyl ring, with dihedral angles of 6.23 (11)° and 4.81 (11)° respectively. The dihedral angle between the two thiazolyl rings is 10.39 (8)°.

The bonding parameters illustrate that C8—C9 and C10—C11 bonds in the thiophenyl ring are localized double bonds (1.354 (5) and 1.358 (4) Å respectively), as are the N1—C7 and N2—C12 bonds (1.286 (4) and 1.308 (4) Å respectively).

Taking into account merely interactions with hydrogen-acceptor distances at least 0.1 Å shorter than the sum of van der Waals radii, the molecules are linked by weak interactions of the type C16—H16···N1, which lead to the formation of zig-zag-chains along [010] (see Fig. 2). The shortest distance in the parallel stacking of the molecules is 3.6371 (17) Å, observed for the planes through the thiophenyl ring and the phenyl ring (C13 to C18) of a neighboring molecule.

### Experimental

All chemicals used (reagent grade) were commercially available. A mass of 0.281 g (0.0020 mol) of 2,5-thiophenedicarboxaldehyde was dissolved in methanol (20 cm^3^), and 0.502 g (0.0040 mol) of 2-aminothiophenol was added with stirring. The mixture was heated under reflux for an hour, then cooled to room temperature and filtered. After standing at 0°C for 24 h, a yellow precipitate was collected. Recrystallization from ethanol gave yellow needles (0.378 g, 54 %), with the formulation C_18_H_10_N_2_S_3_ and suitable for X-ray analysis. *M*.p. 203–206 °C.

### Refinement

The C-bound H atoms were positioned geometrically (0.95 Å for CH) and treated as riding on their parent atoms with U_iso_(H) = 1.2U_eq_(C).

### Crystal data

**Table:**

C_18_H_10_N_2_S_3_	*F*(000) = 720
*M**_r_* = 350.48	*D*_x_ = 1.521 (1) Mg m^−^^3^
Monoclinic, *P*2_1_/*c*	Mo *K*α radiation, λ = 0.71073 Å
*a* = 15.7297 (14) Å	Cell parameters from 1275 reflections
*b* = 8.2396 (5) Å	θ = 4.2–26.2°
*c* = 12.8160 (12) Å	µ = 0.48 mm^−^^1^
β = 112.872 (11)°	*T* = 200 K
*V* = 1530.4 (2) Å^3^	Needles, yellow
*Z* = 4	0.34 × 0.15 × 0.01 mm

### Data collection

**Table:**

Oxford XCalibur diffractometer	3086 independent reflections
Radiation source: fine-focus sealed tube	1452 reflections with *I* > 2σ(*I*)
graphite	*R*_int_ = 0.053
Detector resolution: 15.9809 pixels mm^-1^	θ_max_ = 26.3°, θ_min_ = 4.2°
ω scans	*h* = −19→19
Absorption correction: multi-scan (*CrysAlis PRO*; Oxford Diffraction, 2009)	*k* = −9→10
*T*_min_ = 0.839, *T*_max_ = 1.000	*l* = −9→15
5789 measured reflections	

### Refinement

**Table:**

Refinement on *F*^2^	Primary atom site location: structure-invariant direct methods
Least-squares matrix: full	Secondary atom site location: difference Fourier map
*R*[*F*^2^ > 2σ(*F*^2^)] = 0.043	Hydrogen site location: inferred from neighbouring sites
*wR*(*F*^2^) = 0.051	H-atom parameters constrained
*S* = 0.72	*w* = 1/[σ^2^(*F*_o_^2^) + (0.006*P*)^2^] where *P* = (*F*_o_^2^ + 2*F*_c_^2^)/3
3086 reflections	(Δ/σ)_max_ < 0.001
208 parameters	Δρ_max_ = 0.29 e Å^−^^3^
0 restraints	Δρ_min_ = −0.30 e Å^−^^3^

### Fractional atomic coordinates and isotropic or equivalent isotropic displacement parameters (Å^2^)

**Table:**

	*x*	*y*	*z*	*U*_iso_*/*U*_eq_	
S1	0.19188 (5)	−0.01268 (9)	0.55789 (7)	0.0372 (2)	
S2	0.40390 (5)	0.14749 (8)	0.43819 (7)	0.0294 (2)	
S3	0.65397 (5)	0.41064 (8)	0.64495 (7)	0.0351 (2)	
N1	0.22730 (15)	−0.0478 (2)	0.3780 (2)	0.0306 (7)	
N2	0.57525 (15)	0.3239 (2)	0.4337 (2)	0.0258 (6)	
C1	0.11335 (19)	−0.1200 (3)	0.4461 (3)	0.0294 (8)	
C2	0.1446 (2)	−0.1266 (3)	0.3573 (3)	0.0299 (8)	
C3	0.0918 (2)	−0.2090 (3)	0.2584 (3)	0.0393 (9)	
H3	0.1119	−0.2156	0.1975	0.047*	
C4	0.0108 (2)	−0.2802 (3)	0.2499 (3)	0.0421 (9)	
H4	−0.0250	−0.3369	0.1825	0.051*	
C5	−0.0200 (2)	−0.2717 (3)	0.3369 (3)	0.0436 (9)	
H5	−0.0766	−0.3220	0.3284	0.052*	
C6	0.0300 (2)	−0.1917 (3)	0.4350 (3)	0.0383 (9)	
H6	0.0084	−0.1851	0.4946	0.046*	
C7	0.25959 (18)	0.0157 (3)	0.4775 (3)	0.0252 (7)	
C8	0.34453 (18)	0.1056 (3)	0.5237 (3)	0.0233 (7)	
C9	0.38812 (19)	0.1676 (3)	0.6290 (3)	0.0321 (8)	
H9	0.3656	0.1568	0.6875	0.039*	
C10	0.46999 (19)	0.2493 (3)	0.6426 (2)	0.0292 (8)	
H10	0.5091	0.2988	0.7115	0.035*	
C11	0.48737 (18)	0.2503 (3)	0.5468 (2)	0.0242 (7)	
C12	0.56531 (19)	0.3212 (3)	0.5304 (3)	0.0252 (8)	
C13	0.70982 (19)	0.4587 (3)	0.5569 (3)	0.0278 (8)	
C14	0.6576 (2)	0.4025 (3)	0.4477 (3)	0.0280 (8)	
C15	0.6891 (2)	0.4295 (3)	0.3612 (3)	0.0372 (8)	
H15	0.6546	0.3929	0.2862	0.045*	
C16	0.7711 (2)	0.5105 (3)	0.3873 (3)	0.0401 (9)	
H16	0.7925	0.5313	0.3287	0.048*	
C17	0.8235 (2)	0.5627 (3)	0.4957 (3)	0.0392 (9)	
H17	0.8806	0.6163	0.5109	0.047*	
C18	0.79350 (19)	0.5377 (3)	0.5819 (3)	0.0370 (9)	
H18	0.8291	0.5735	0.6567	0.044*	

### Atomic displacement parameters (Å^2^)

**Table:**

	*U*^11^	*U*^22^	*U*^33^	*U*^12^	*U*^13^	*U*^23^
S1	0.0338 (5)	0.0475 (5)	0.0327 (6)	−0.0084 (4)	0.0156 (4)	−0.0013 (4)
S2	0.0278 (4)	0.0338 (4)	0.0285 (5)	−0.0019 (4)	0.0131 (4)	−0.0010 (4)
S3	0.0355 (5)	0.0414 (5)	0.0292 (6)	−0.0081 (4)	0.0136 (4)	−0.0030 (4)
N1	0.0267 (16)	0.0288 (16)	0.039 (2)	−0.0070 (12)	0.0154 (14)	−0.0019 (12)
N2	0.0235 (14)	0.0293 (14)	0.0270 (18)	−0.0032 (12)	0.0124 (13)	0.0000 (12)
C1	0.0301 (18)	0.0262 (18)	0.031 (2)	0.0001 (15)	0.0110 (16)	0.0036 (14)
C2	0.0319 (19)	0.0250 (18)	0.031 (2)	0.0034 (15)	0.0107 (17)	0.0043 (15)
C3	0.046 (2)	0.045 (2)	0.032 (2)	−0.0112 (17)	0.0211 (19)	−0.0050 (17)
C4	0.037 (2)	0.047 (2)	0.037 (3)	−0.0182 (17)	0.0080 (19)	−0.0047 (17)
C5	0.035 (2)	0.048 (2)	0.048 (3)	−0.0127 (17)	0.016 (2)	0.0046 (19)
C6	0.033 (2)	0.046 (2)	0.039 (3)	−0.0050 (17)	0.0173 (19)	0.0065 (18)
C7	0.0272 (19)	0.0201 (16)	0.031 (2)	0.0017 (15)	0.0142 (17)	0.0023 (16)
C8	0.0214 (17)	0.0226 (16)	0.028 (2)	−0.0018 (14)	0.0125 (16)	0.0004 (15)
C9	0.0338 (19)	0.0379 (18)	0.030 (2)	−0.0021 (15)	0.0182 (18)	0.0067 (16)
C10	0.0250 (18)	0.0350 (18)	0.025 (2)	−0.0007 (15)	0.0066 (16)	−0.0008 (15)
C11	0.0260 (18)	0.0194 (16)	0.025 (2)	0.0006 (14)	0.0079 (17)	0.0001 (14)
C12	0.0295 (19)	0.0170 (16)	0.029 (2)	0.0018 (14)	0.0107 (17)	0.0010 (14)
C13	0.0241 (18)	0.0270 (18)	0.036 (2)	0.0003 (14)	0.0154 (17)	0.0032 (15)
C14	0.0318 (19)	0.0253 (17)	0.029 (2)	0.0035 (15)	0.0148 (17)	0.0016 (15)
C15	0.045 (2)	0.0423 (19)	0.029 (2)	−0.0049 (17)	0.0191 (18)	−0.0056 (16)
C16	0.041 (2)	0.0410 (19)	0.051 (3)	−0.0025 (17)	0.033 (2)	0.0007 (19)
C17	0.031 (2)	0.0324 (18)	0.056 (3)	−0.0053 (16)	0.020 (2)	−0.0017 (18)
C18	0.0306 (19)	0.039 (2)	0.033 (2)	−0.0048 (15)	0.0033 (17)	−0.0002 (16)

### Geometric parameters (Å, °)

**Table:**

S1—C1	1.728 (3)	C6—H6	0.9500
S1—C7	1.761 (3)	C7—C8	1.438 (3)
S2—C11	1.720 (3)	C8—C9	1.353 (3)
S2—C8	1.729 (3)	C9—C10	1.403 (3)
S3—C13	1.725 (3)	C9—H9	0.9500
S3—C12	1.747 (3)	C10—C11	1.357 (3)
N1—C7	1.286 (3)	C10—H10	0.9500
N1—C2	1.384 (3)	C11—C12	1.445 (3)
N2—C12	1.308 (3)	C13—C18	1.389 (3)
N2—C14	1.397 (3)	C13—C14	1.398 (4)
C1—C6	1.394 (3)	C14—C15	1.396 (4)
C1—C2	1.404 (4)	C15—C16	1.374 (3)
C2—C3	1.392 (3)	C15—H15	0.9500
C3—C4	1.368 (4)	C16—C17	1.380 (4)
C3—H3	0.9500	C16—H16	0.9500
C4—C5	1.379 (4)	C17—C18	1.375 (4)
C4—H4	0.9500	C17—H17	0.9500
C5—C6	1.366 (4)	C18—H18	0.9500
C5—H5	0.9500		
			
?···?	?		
			
C1—S1—C7	89.01 (14)	C8—C9—H9	123.5
C11—S2—C8	90.91 (14)	C10—C9—H9	123.5
C13—S3—C12	89.42 (14)	C11—C10—C9	112.8 (3)
C7—N1—C2	111.1 (3)	C11—C10—H10	123.6
C12—N2—C14	109.4 (2)	C9—C10—H10	123.6
C6—C1—C2	120.8 (3)	C10—C11—C12	127.9 (3)
C6—C1—S1	129.9 (3)	C10—C11—S2	111.7 (2)
C2—C1—S1	109.2 (2)	C12—C11—S2	120.3 (2)
N1—C2—C3	125.9 (3)	N2—C12—C11	124.4 (3)
N1—C2—C1	115.2 (3)	N2—C12—S3	116.0 (2)
C3—C2—C1	118.9 (3)	C11—C12—S3	119.5 (2)
C4—C3—C2	119.3 (3)	C18—C13—C14	121.4 (3)
C4—C3—H3	120.4	C18—C13—S3	129.3 (3)
C2—C3—H3	120.4	C14—C13—S3	109.2 (2)
C3—C4—C5	121.5 (3)	C15—C14—N2	124.7 (3)
C3—C4—H4	119.3	C15—C14—C13	119.4 (3)
C5—C4—H4	119.3	N2—C14—C13	115.9 (3)
C6—C5—C4	120.8 (3)	C16—C15—C14	118.3 (3)
C6—C5—H5	119.6	C16—C15—H15	120.9
C4—C5—H5	119.6	C14—C15—H15	120.9
C5—C6—C1	118.6 (3)	C15—C16—C17	122.2 (3)
C5—C6—H6	120.7	C15—C16—H16	118.9
C1—C6—H6	120.7	C17—C16—H16	118.9
N1—C7—C8	124.3 (3)	C18—C17—C16	120.3 (3)
N1—C7—S1	115.5 (2)	C18—C17—H17	119.8
C8—C7—S1	120.2 (2)	C16—C17—H17	119.8
C9—C8—C7	129.4 (3)	C17—C18—C13	118.3 (3)
C9—C8—S2	111.5 (2)	C17—C18—H18	120.8
C7—C8—S2	119.1 (2)	C13—C18—H18	120.8
C8—C9—C10	113.0 (3)		
			
C7—S1—C1—C6	−178.8 (3)	C9—C10—C11—C12	−179.3 (2)
C7—S1—C1—C2	0.3 (2)	C9—C10—C11—S2	−0.9 (3)
C7—N1—C2—C3	−179.5 (3)	C8—S2—C11—C10	0.7 (2)
C7—N1—C2—C1	0.5 (3)	C8—S2—C11—C12	179.2 (2)
C6—C1—C2—N1	178.7 (2)	C14—N2—C12—C11	179.5 (2)
S1—C1—C2—N1	−0.5 (3)	C14—N2—C12—S3	−0.6 (3)
C6—C1—C2—C3	−1.3 (4)	C10—C11—C12—N2	−176.7 (3)
S1—C1—C2—C3	179.5 (2)	S2—C11—C12—N2	5.1 (4)
N1—C2—C3—C4	−179.5 (3)	C10—C11—C12—S3	3.4 (4)
C1—C2—C3—C4	0.5 (4)	S2—C11—C12—S3	−174.81 (13)
C2—C3—C4—C5	0.3 (5)	C13—S3—C12—N2	0.6 (2)
C3—C4—C5—C6	−0.3 (5)	C13—S3—C12—C11	−179.5 (2)
C4—C5—C6—C1	−0.6 (5)	C12—S3—C13—C18	−179.6 (3)
C2—C1—C6—C5	1.4 (4)	C12—S3—C13—C14	−0.4 (2)
S1—C1—C6—C5	−179.6 (2)	C12—N2—C14—C15	−178.8 (3)
C2—N1—C7—C8	−180.0 (2)	C12—N2—C14—C13	0.2 (3)
C2—N1—C7—S1	−0.3 (3)	C18—C13—C14—C15	−1.5 (4)
C1—S1—C7—N1	0.0 (2)	S3—C13—C14—C15	179.3 (2)
C1—S1—C7—C8	179.7 (2)	C18—C13—C14—N2	179.4 (2)
N1—C7—C8—C9	−174.9 (3)	S3—C13—C14—N2	0.2 (3)
S1—C7—C8—C9	5.4 (4)	N2—C14—C15—C16	179.2 (2)
N1—C7—C8—S2	5.6 (4)	C13—C14—C15—C16	0.3 (4)
S1—C7—C8—S2	−174.02 (14)	C14—C15—C16—C17	1.2 (4)
C11—S2—C8—C9	−0.3 (2)	C15—C16—C17—C18	−1.4 (5)
C11—S2—C8—C7	179.2 (2)	C16—C17—C18—C13	0.1 (4)
C7—C8—C9—C10	−179.6 (2)	C14—C13—C18—C17	1.3 (4)
S2—C8—C9—C10	−0.1 (3)	S3—C13—C18—C17	−179.7 (2)
C8—C9—C10—C11	0.7 (4)		

### Hydrogen-bond geometry (Å, °)

**Table:**

*D*—H···*A*	*D*—H	H···*A*	*D*···*A*	*D*—H···*A*
C16—H16···N1^i^	0.95	2.63	3.444 (4)	144

Symmetry codes: (i) −*x*+1, *y*+1/2, −*z*+1/2.
